# Hippocampal Shape and Volume Changes with Antipsychotics in Early Stage Psychotic Illness

**DOI:** 10.3389/fpsyt.2012.00096

**Published:** 2012-11-12

**Authors:** Daniel Mamah, Michael P. Harms, Deanna Barch, Martin Styner, Jeffrey A. Lieberman, Lei Wang

**Affiliations:** ^1^Department of Psychiatry, Washington UniversitySt. Louis, MO, USA; ^2^Department of Psychology, Washington UniversitySt. Louis, MO, USA; ^3^Department of Anatomy and Neurobiology, Washington UniversitySt. Louis, MO, USA; ^4^Neurodevelopmental Disorders Research Center, University of North CarolinaChapel Hill, NC, USA; ^5^Department of Psychiatry, Columbia University Medical CenterNew York, NY, USA; ^6^Department of Psychiatry and Behavioral Sciences, Northwestern University Feinberg School of MedicineChicago, IL, USA

**Keywords:** schizophrenia, psychosis, hippocampus, olanzapine, haloperidol, MRI

## Abstract

Progression of hippocampal shape and volume abnormalities has been described in psychotic disorders such as schizophrenia. However it is unclear how specific antipsychotic medications influence the development of hippocampal structure. We conducted a longitudinal, randomized, controlled, multisite, double-blind study involving 14 academic medical centers (United States 11, Canada 1, Netherlands 1, and England 1). One hundred thirty-four first-episode psychosis patients (receiving either haloperidol [HAL] or olanzapine [OLZ]) and 51 healthy controls were followed for up to 104 weeks using magnetic resonance imaging and large-deformation high-dimensional brain mapping of the hippocampus. Changes in hippocampal volume and shape metrics (i.e., percentage of negative surface vertex slopes, and surface deformation) were evaluated. Mixed-models analysis did not show a significant group-by-time interaction for hippocampal volume. However, the cumulative distribution function of hippocampal surface vertex slopes showed a notable left shift with HAL treatment compared to OLZ treatment and to controls. OLZ treatment was associated with a significantly lower percentage of “large magnitude” negative surface vertex slopes compared to HAL treatment (*p* = 0.004). Surface deformation maps however did not localize any hippocampal regions that differentially contracted over time with OLZ treatment, after FDR correction. These results indicate that surface analysis provides supplementary information to volumetry in detecting differential treatment effects of the hippocampus. Our results suggest that OLZ is associated with less longitudinal hippocampal surface deformation than HAL, however the hippocampal regions affected appear to be variable across patients.

## Introduction

Abnormalities of the hippocampus are widely implicated as contributing to the pathophysiology of schizophrenia. The hippocampus is involved in encoding explicit long-term memory, both episodic and semantic, which are often found to be dysfunctional in patients with psychotic disorders (Goldberg et al., 1998; Boyer et al., 2007). Structural abnormalities of the hippocampus have also been noted in psychosis (Mamah et al., 2009). For example, the majority of *in vivo* imaging studies show hippocampal volume reductions in schizophrenia patients compared to healthy controls (Lawrie and Abukmeil, 1998; Nelson et al., 1998; Wright et al., 2000; Honea et al., 2005).

Computational image analysis methods have several advantages over traditional volumetric approaches in evaluating brain structures. They can isolate abnormalities locally within a structure and identify more subtle abnormalities potentially associated with neuropathy. Computerized methods have been used to characterize surface features of regions of interest in three-dimensional space (Thompson et al., 2000; Ashburner et al., 2003). Some of these methods have found regional alterations in hippocampal shape, with anterior-lateral regions affected, in schizophrenia (Haller et al., 1996; Csernansky et al., 1998, 2002; Wang et al., 2001; Mamah et al., 2010). Other shape analysis tools however found abnormalities in other hippocampal regions (Shenton et al., 2002; Styner et al., 2004). In spite of differences in results between laboratories, the shape metrics reported by the various groups show promise for clarifying the nature of hippocampal morphological changes in schizophrenia.

Several lines of evidence suggest a progressive neuropathologic process that underlies the deteriorating clinical course of schizophrenia (Loebel et al., 1992; Davis, 1999; Lieberman, 1999). Longitudinal imaging studies of the hippocampus in schizophrenia have often revealed progression of volume decrease in the hippocampus (Velakoulis et al., 2000; Szeszko et al., 2002; Joyal et al., 2003). Using *Large-Deformation High-Dimensional Brain Mapping* (HDBM-LD), our group found significantly abnormal hippocampal shape change (over 2 years) in schizophrenia in the absence of volumetric abnormalities (Wang et al., 2008), suggesting that shape analysis is complementary to volumetry in assessing longitudinal changes.

It remains unclear to what degree antipsychotic treatment confounds longitudinal imaging studies of hippocampal morphology in patients. Preclinical studies have suggested that specific atypical antipsychotic drugs may have pharmacologic properties that could produce neurotrophic, neurogenetic, or neuroprotective effects (Wakade et al., 2002; Bai et al., 2003; Halim et al., 2004). A limitation of the existing studies on effects of antipsychotics on brain structure is that they have often investigated patients treated for many years, with different types of antipsychotic drugs. This makes it difficult to disentangle which brain changes are due to a specific class of antipsychotics, and which are due to the illness and its progression (Dazzan and Murray, 1999). Therefore, these questions can be better addressed by investigating subjects at the initial stages of psychosis, when treatment would have occurred for only a short time. The older, “typical” antipsychotics, such as HAL, act on the dopaminergic system by blocking the dopamine type 2 (D2) receptors in mesolimbic areas (Carlsson, 1978). Newer, “atypical” antispychotics have lower affinity and occupancy for the dopaminergic receptors, and a high degree of occupancy of the serotoninergic receptors 5-HT2A (Meltzer et al., 1999). Differences in receptor affinities between the two general drug classes have been linked to greater effectiveness of atypical antipsychotics on cognitive dysfunction and negative symptoms in schizophrenia patients (Abdul-Monim et al., 2003; Karow et al., 2006; Meltzer and Sumiyoshi, 2008; He et al., 2009), although other studies have showed no such advantages (Rollnik et al., 2002; White et al., 2006).

In patients with first-episode psychosis, Lieberman et al. (2005) found that HAL-treated patients exhibited significant decreases in cortical gray matter volume, whereas patients treated with the atypical antipsychotic olanzapine (OLZ) did not. In this study, we investigated the longitudinal effects of OLZ and HAL treatment on hippocampal volume and shape, using most of the same subjects previously studied (Lieberman et al., 2005). We hypothesized that OLZ would lead to less global and regional hippocampal volume decrease compared to HAL.

## Materials and Methods

This longitudinal study was conducted at 14 academic medical centers (11 in the United States, 1 in Canada, 1 in the Netherlands, and 1 in England; Lieberman et al., 2005).

### Participants

Table 1 shows participant profiles in each group. One hundred thirty-four first-episode psychotic patients (PSY) and 51 healthy controls (CON) were included in the study. Patients included those who presented for clinical services. Inclusion criteria included: (1) diagnosis of schizophrenia, schizophreniform, or schizoaffective disorder according to DSM-IV criteria (as assessed with the Structured Clinical Interview for DSM-IV, Research Version (First et al., 2002), (2) age 16–40 years, (3) onset of psychotic symptoms before age 35 years, and (4) premorbid IQ of 70 or more. Exclusion criteria included (1) previous antipsychotic drug treatment of more than 16 cumulative weeks, or treatment with clozapine at any time (2) current substance dependence (except caffeine and nicotine) within 1 month before study entry, and (3) treatment with anticonvulsants, benzodiazepines (except as allowed for agitation and control of extrapyramidal symptoms), antidepressants, psychostimulants, or other antipsychotic drugs at study entry. Healthy volunteers matched to the patients’ demographic characteristics were ascertained from respondents to advertisements.

**Table 1:** **Demographic and clinical profiles**.

Characteristics	Control *n* = 51	Olanzapine *n* = 67	Haloperidol *n* = 67	*p*-Value	
				Olz vs. Hal	Scz vs. Con
Age	25.0 (3.9)	23.7 (4.6)	24.3 (4.7)	0.46	0.16
Gender – *N* (%)
Female	16 (31.4)	13 (19.4)	8 (11.9)	0.23	0.02*
Male	35 (68.6)	54 (80.6)	59 (88.1)		
Race – *N* (%)
African American	15 (29.4)	23 (34.3)	29 (47.5)	0.44	0.23
Asian	0	1 (1.5)	0		
Caucasian	30 (58.8)	36 (53.7)	28 (45.9)		
East Asian	4 (7.8)	1 (1.5)	1 (1.6)		
Hispanic	1 (2.0)	3 (4.5)	3 (4.9)		
Other	1 (2.0)	3 (4.5)	0		
Illness duration (weeks)	–	57.1 (52.0)	78.4 (63.0)	0.034*	n/a
Diagnosis–*N* (%)
Schizophrenia, disorganized	–	3 (4.4)	2 (3.0)	0.48	n/a
Schizophrenia, paranoid	–	25 (37.3)	31 (49.3)		
Schizophrenia, undifferentiated	–	11 (16.4)	15 (22.4)		
Schizophreniform disorder	–	20 (29.9)	15 (22.4)		
Schizoaffective disorder	–	8 (11.9)	4 (6.0)		
Time point–*N* (% of week 0)
Baseline (week 0)	51 (100)	67 (100)	67 (100)	n/a	n/a
Week 12	47 (92.2)	60 (89.6)	61 (91.0)	n/a	n/a
Week 24	–	56 (83.6)	40 (59.7)	n/a	n/a
Week 52	43 (84.3)	37 (55.2)	30 (44.8)	n/a	n/a
Week 104	–	24 (35.8)	11 (16.4)	n/a	n/a

*Values are means (standard deviation) unless stated otherwise*.

*Statistically significant (*p* < 0.05) between group differences.

*n/a = not applicable*.

### Study design and procedures

Patients were randomized to double-blind treatment with OLZ, 5–20 mg/day, or HAL, 2–20 mg/day, for up to 104 weeks. Permitted concomitant medications (for no more than 21 days) included chloral hydrate, lorazepam, or diazepam, for the management of agitation, general behavior disturbances, and/or insomnia. If clinically important extrapyramidal symptoms emerged, anticholinergic medication was also permitted. Antidepressants (except fluoxetine hydrochloride) and/or mood stabilizers were not allowed in the first 12 weeks of the study.

### Image acquisition and preprocessing

Scans used for the study included the majority of those assessed in a prior longitudinal study of brain volume (Lieberman et al., 2005). Briefly, participants were assessed using MRI at weeks 0 (baseline), 12, 24, 52, and 104. All MRI studies were performed on 1.5T MRI systems. Six of the eight imaging sites used Signa scanners (General Electric Co., Milwaukee, WI, USA), and two sites used a Gyroscan scanner (Philips Medical Systems, Best, the Netherlands). The imaging protocol included 3-dimensional T1-weighted, inversion recovery-prepared spoiled gradient-recalled acquisition in steady state images (0.94 mm × 0.94 mm × 1.50 mm, axial direction) and contiguous proton density and T2-weighted fast spin-echo images (0.94 mm × 0.94 mm × 3.00 mm, axial slicing direction). Quality control scans were performed twice a month on each MRI system with standardized imaging phantoms. Rigorous standardization and quality control procedures were used and reliability of the measurements across sites was established and maintained throughout the study (Styner et al., 2002).

### Surface generation

Hippocampal surface generation of baseline scans was done by HDBM-LD from a neuroanatomical template (Haller et al., 1997; Miller et al., 1997a; Csernansky et al., 2004). An MR scan collected from a healthy comparison subject was used to construct a neuroanatomical template (Csernansky et al., 1998, 2002; Wang et al., 2008). In the construction of this template the right hippocampus was manually outlined by expert consensus using atlas (Mai et al., 1997) guidelines. A set of landmarks was developed for placement within the hippocampus in MR scans of the template and each study subject. Transformation of the template MR scan onto the MR scan of study subjects (“subject scan”) occurred in two steps. First, the template scan was coarsely aligned to the left and right sides of each subject scan using the landmarks. Second, HDBM-LD was used to determine the transformation between template and subject scan (Haller et al., 1997). To derive a surface for each hippocampus, a triangulated graph was first superimposed onto the surface of the hippocampus in the right hemisphere of the template scan. This surface was then carried along as the template scan was transformed to match the left and right sides of each of the subject scans. The reliability of this process is comparable to manual outlining by experts for defining the neuroanatomical boundaries of the hippocampus (Haller et al., 1997; Csernansky et al., 1998). To map the surfaces at follow-up, baseline and follow-up scans were first registered using a nine-parameter affine transformation to adjust for changes in head position and scanner-drift (Freeborough et al., 1996). Next, HDBM-LD was used in neuroanatomic regions immediately surrounding the structures of interest, at twice the native-scan resolution (Wang et al., 2008).

Atlas based segmentation of total cortical gray matter (Gouttard et al., 2007) were done to allow for cortical volume to be used as a covariate in baseline hippocampal volume analyses.

### Baseline analysis of hippocampal volume and shape

Left and right baseline volumes were entered into a repeated-measures analysis of variance (RM-ANOVA) model, with group as the main effect, and hemisphere as a repeated factor.

To quantify surface shape, we first applied principal components analysis (PCA) to the baseline right and left surfaces for dimensionality reduction (Csernansky et al., 2002; Wang et al., 2008). The first 20 principal components (PC) accounted for more than 90% of total surface variance. To evaluate baseline differences in overall hippocampal shape, the 20 PC scores were entered into a RM-MANOVA with diagnostic group as the main effect, and hemisphere as a repeated factor.

### Longitudinal analysis of hippocampal volume and shape

To evaluate longitudinal effects on hippocampus volume, for each subject, slopes of volume change over the time were calculated using linear least squares across all available time points (see Table 1 for distribution of available time points). Slopes were then compared using RM-ANOVA with hemisphere as a repeated factor. As a secondary longitudinal analysis, mixed-models analyses of hippocampal volume with time as within subjects factor were performed.

Evaluation of the longitudinal effect on hippocampal shape was done using *the percentage of negative surface vertex slopes* and *the percentage of* *“large magnitude” surface vertex slopes*. These measures were computed across vertices individually in each subject, using the slope map for each subject. Large magnitude surface vertex slopes (i.e., “very negative slopes” and “very positive slopes”) were evaluated since these may differ between groups, despite a similar proportion of negative slopes. “Very negative slopes” were defined as slopes with values more negative than 1 SD below the mean slope of controls. “Very positive slopes” had values more positive that 1 SD above the mean slope of controls. These measures were then were compared across subjects using ANOVA, since the distribution of values approximated normality (Shapiro–Wilk test, *p* > 0.05).

To visualize potential regional effects, slope maps were computed on the hippocampi of individual subjects, using slope values at each surface vertex (13,222 over both hippocampi). Values at each vertex were then averaged across subjects to generate mean group hippocampal slope maps, which were compared using the Mann–Whitney *U* test. To correct for multiple comparisons across the multiple hippocampal surface vertices, false discovery rate (FDR) thresholding was applied in which the *p*-value maps were thresholded to yield an FDR of 5% (i.e., *q* = 0.05). For visualization of the mean slope maps, slope directions were color-coded, with warmer (i.e., red-orange) colors depicting positive slopes, and cooler colors (i.e., purple-blue) depicting negative slopes.

## Results

### Baseline volume analyses

At baseline, volume in mm^3^ (and standard deviation) of the left hippocampus was 2,252 (304) and right hippocampus was 2,696 (374) in psychotic subjects (PSY), while left hippocampus was 2,528 (318) and right hippocampus was 3,010 (313) in controls. There was a significant group effect [*F*(1,183) = 33.2, *p* < 0.0001]. Across all subjects, there was a significant effect of hemisphere [right > left; *F*(1,183) = 531.5, *p* < 0.0001], but no significant group-by-hemisphere interaction. Results of group comparisons were similar after controlling for gender [*F*(1,182) = 40.8, *p* < 0.0001] or total gray matter volume [*F*(1,182) = 41.2, *p* < 0.0001]. Additionally controlling for illness duration did not affect volume comparisons. Baseline comparisons of the hippocampal volume in PSY did not show any significant differences between the groups that were subsequently treated with OLZ vs. HAL on the left (*p* = 0.99) or right (*p* = 0.65).

At baseline, volume (mm^3^) of total brain gray matter was 691,758 (72,941) in PSY and 705,594 (69,608) in controls. There was no significant group effect of total gray matter [*F*(1,183) = 1.4; *p* = 0.24], however correction for gender resulted in a significant group effect [CON > PSY; *F*(1,182) = 5.7; *p* = 0.02].

### Baseline hippocampal shape analyses

**Figure 1:**
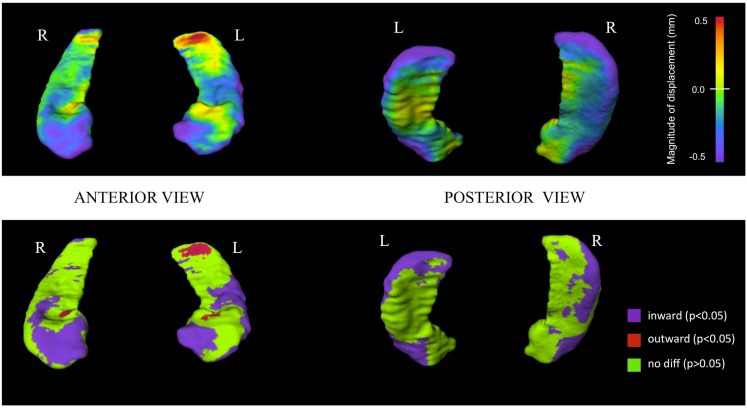
**Hippocampal surface maps showing baseline structural differences between first-episode psychosis patients and controls**. Hippocampal surface generation of baseline scans was done by high-dimensional brain mapping (HDBM-LD) from a neuroanatomical template (see Materials and Methods). Groups were compared at each of 13,222 corresponding vertices on the hippocampal surface. *Top row:* structural differences are shown using a continuous color-coded scale. Purple/blue shading denotes regions of inward surface displacement (i.e., surface contraction) in first-episode psychosis patients compared to controls. Red/orange shading denotes regions of outward displacement (i.e., surface expansion). *Bottom row:* structural differences shown using a color-coded scale thresholded at statistical significance. Purple regions show statistically significant (*p* < 0.05) inward displacement in psychotic patients compared to controls. Red regions show statistically significant (*p* < 0.05) outward displacement in psychotic patients compared to controls. Green regions indicate statistically similar group values. Surface vertex differences are thresholded at a false discovery rate (FDR) of 5% (i.e., *q* = 0.05) applied to Mann–Whitney *U* test based *p*-values.

A comparison of hippocampal shape in PSY and CON done using a MANOVA with the first 20 shape principal component scores (eigenvectors) showed significant differences on the left (Wilks = 0.69; *p* < 0.0001) and on the right (Wilks = 0.66; *p* < 0.0001). Controlling for gender or illness duration did not change results of the analysis. Surface maps depicting hippocampal shape in PSY are shown in Figure 1. Visual observation shows inward deformation particularly of the head and lateral regions (extending into the tail) of the hippocampus in PSY, compared to CON. There was also a region of slight outward deformation of the postero-dorsal surface of the left hippocampus tail in PSY.

### Longitudinal hippocampal volume analyses

#### Slope comparisons

Left hippocampal volume slopes in mm^3^ per week were −0.80 (2.8) in OLZ, −1.94 (5.4) in HAL, and −0.45 (1.8) in CON. Right hippocampal slopes were −0.93 (2.0) in OLZ, −1.58 (6.3) in HAL, and −0.47 (2.1) in CON. There was no significant overall group effect [*F*(2,182) = 1.9, *p* = 0.15], hemisphere effect (*p* = 0.7), or group-by-hemisphere interaction (*p* = 0.5).

#### Mixed-models analysis

Longitudinal analysis comparing CON, OLZ, and HAL on hippocampal volume showed a significant time effect of the hippocampus on the left [*F*(4,399) = 9.8, *p* < 0.0001] and right [*F*(4,399) = 13.5, *p* < 0.0001] across groups, but no significant group-by-time interaction (left: *p* = 0.76; right: *p* = 0.56).

### Longitudinal shape analyses

#### Cumulative distribution of surface vertex slopes

**Figure 2:**
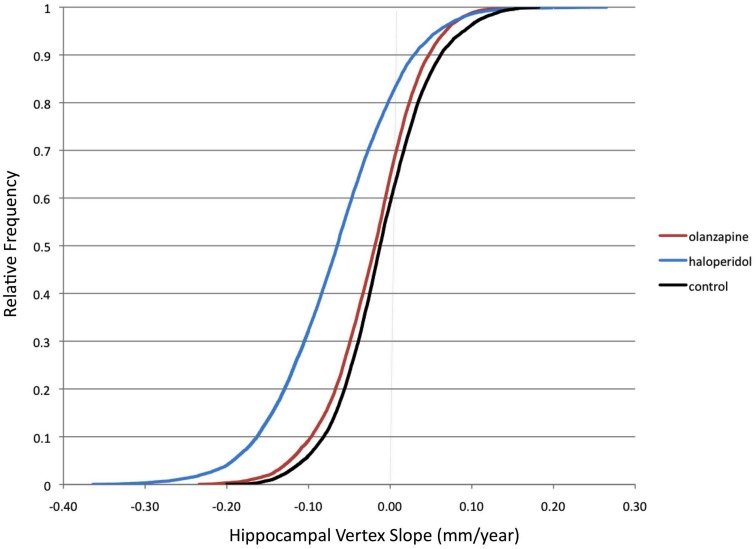
**Cumulative distribution functions of hippocampal surface vertices**. Group distributions of mean slope (mm/year) across the 13,222 hippocampal surface vertices (left and right combined) are shown. Slopes were calculated using all available visits (up to a maximum follow-up of 104 weeks). Black = Healthy controls. Red = Olanzapine-treated patients. Blue = Haloperidol-treated patients.

Figure 2 shows the cumulative distribution functions of the mean slope map for each subject group. (The distribution is across the 13,222 hippocampal surface vertices). As can be seen in this Figure, the distributions of surface vertex slopes in HAL were shifted toward more negative values compared to CON, while OLZ had an intermediate distribution. Differences between HAL and CON also appeared to be greater at more negative slopes. The group averages of the vertex slopes (averaged across all vertices, in mm/year) were −0.011 (0.057) for CON, −0.022 (0.057) for OLZ, and −0.066 (0.077) for HAL.

We used two approaches to quantify this effect. First, we examined the percentage of negative slopes in each group (see Table 2), using an ANOVA with group, and hemisphere as factors. This ANOVA did not show significant group differences [*F*(2,182) = 2.2, *p* = 0.12], and there were no significant hemisphere effects or group *x* hemisphere interactions. Second, we then evaluated the percentage of hippocampal surface vertex slopes more negative in magnitude than 1 standard deviation (SD) below or above the mean slope in controls (see Table 2). This ANOVA showed a highly significant main effect of group [*F*(2,182) = 6.5, *p* = 0.0019]. *Post hoc* analyses showed significant differences between HAL vs. OLZ (*p* = 0.004), and HAL vs. CON (*p* = 0.003), but no significant differences between OLZ vs. CON (*p* = 0.8). There was no significant effect of hemisphere and no significant group × hemisphere interaction.

**Table 2:** **Statistical analysis of surface point slopes across groups**.

Analysis	Group value (SD)			F or H	*p*
	CON	OLZ	HAL	
**PERCENT NEGATIVE SLOPE:**					
**% Negative Surface Slopes**^a^
Left hippocampus	52.4 (8.3)	53.9 (10.2)	56.6 (12.2)	2.38	0.10
Right hippocampus	52.2 (7.2)	53.5 (10.0)	55.1 (11.6)	1.28	0.28
**% Surface Slopes Negative of 1 SD Below Mean**^b^
Left hippocampus	35.8 (10.2)	35.0 (16.0)	43.3 (16.1)	6.25	0.002*
Right hippocampus	35.4 (8.2)	35.0 (15.1)	42.1 (15.1)	5.59	0.004*
**% Surface Slopes Positive of 1 SD Above Mean**^c^
Left hippocampus	36.1 (9.1)	32.7 (12.8)	34.1 (12.7)	1.15	0.32
Right hippocampus	36.5 (8.2)	33.3 (13.0)	35.7 (12.1)	1.26	0.29

*ANOVA used for analysis of “percent negative slope” comparisons across the three groups. Percent negative slopes’ are given in percentages (standard deviation)*.

*^a^Percentage of hippocampal surface points with longitudinal slope values <0*.

*^b^Percentage of hippocampal surface points with longitudinal slope values more negative than 1 standard deviation below the average mean surface point value in left and right hemisphere of controls (−0.06798 mm/year)*.

*^c^Percentage of hippocampal surface points with longitudinal slope values more positive than 1 standard deviation above the average mean surface point value in left and right hemisphere of controls (0.04680 mm/year)*.

*Statistically significant (*p* < 0.05) after correcting for multiple comparisons.

#### Surface map generation

**Figure 3:**
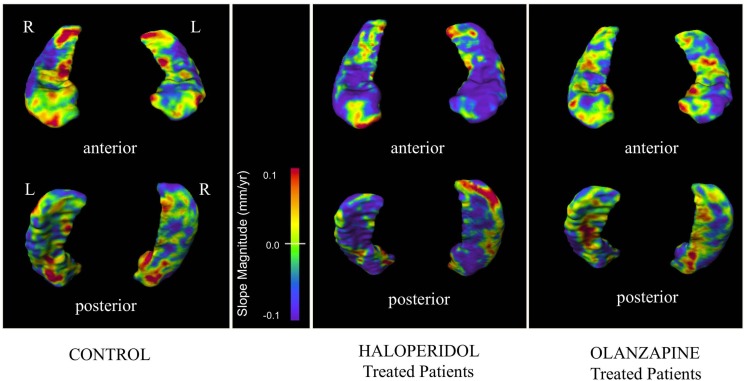
**Hippocampal surface maps showing progression of surface displacement**. Mean slope values (depicting longitudinal structural change over a maximum period of 104 weeks) at each hippocampal surface vertex were calculated in each group. Slope values are color-coded, with purple/blue regions denoting regions of longitudinal surface contraction and red/orange regions denoting regions of longitudinal surface expansion. Surface maps are thresholded between slope values of ±0.1 mm/year.

Visual representations of hippocampus surface vertex slopes are depicted in Figure 3. HAL treatment was associated with large confluent regions of negative longitudinal slopes throughout the hippocampal body, head, and tail. In both CON and OLZ hippocampi, there were fewer negative and more positive slope regions than in HAL hippocampi. In addition, negative slopes appeared lesser in magnitude in CON and OLZ compared to HAL. There were no statistically significant surface vertex slope differences between HAL and OLZ after FDR correction at the 0.05 level.

## Discussion

Our study utilized shape analysis methodology to evaluate the hippocampus in young patients at a very early stage of psychotic illness. We found reduced hippocampal volume in patients, consistent with most previous findings of decreased size of the hippocampus in schizophrenia (Velakoulis et al., 1999, 2006; Gur et al., 2000), and in first-episode psychosis (Whitworth et al., 1998; Sumich et al., 2002; Steen et al., 2006; Vita and de Peri, 2007; Ebdrup et al., 2010). Compared to controls, we found that psychotic patients had hippocampal surface contraction at baseline predominantly on areas of the anterior hippocampus and lateral parts of its body and tail. The pattern of this hippocampal deformation was similar to what we previously found in chronic schizophrenia patients using identical mapping methodology (Csernansky et al., 1998, 2002). Inward deformations along the lateral edges extending toward the tail and an outward deformation on the left dorsal tail, which were observed in our current study, were however not present in our previous chronic patients (Csernansky et al., 1998, 2002), which may suggest greater specificity of anterior hippocampal deformity with more chronic illness.

The clinical significance of regional abnormality within the hippocampus in schizophrenia may be related to regional associations with specific brain regions influencing cognitive function. Functionally, heightened CA3 activity in schizophrenia may generate exaggerated pattern completion memory functions and enhance the production of incorrect associations, which would then produce memories with psychotic content (Corlett et al., 2009; Tamminga et al., 2010). CA3 volumetric enlargement may be supported by our current findings, which showed hippocampal surface expansion in an area of the surface consistent with posterior CA2-CA4 plus dentate gyrus subfields (Csernansky et al., 2005; Wang et al., 2006). We also found that in the hippocampus of those with psychotic disorders, surface contractions occur mainly in regions corresponding to CA1 subfield (located in the anterior and lateral hippocampus) and to a lesser degree, anterior subiculum (Csernansky et al., 2005; Wang et al., 2006). Together, these findings raise the possibility that in first-episode psychosis, reduced nerve fibers, and connections may be present within the CA1 subfield, and the opposite effect to a smaller degree in other regions, possibly the CA3 subfield. Recent human studies have also localized abnormal neuronal activity to the CA1 region in schizophrenia patients and found this to be associated with psychotic symptom severity and a predictor of conversion to syndromal psychosis in prodromal subjects (Schobel et al., 2009).

We found a significant progressive hippocampal volume decrease over time in both first-episode psychosis patients and controls. However, the differences in the rate of progression did not reach statistical significance between groups, consistent with previous studies in first-episode psychosis (DeLisi et al., 1997; Wood et al., 2001; Whitworth et al., 2005). Most longitudinal studies in chronic schizophrenia however indicate progression of volume decrease in the hippocampus (Velakoulis et al., 2000; Szeszko et al., 2002; Joyal et al., 2003), or the anterior hippocampus (Lieberman et al., 2001), although negative results have also been found (Degreef et al., 1991). Thus, our findings may indicate that at the early stage of schizophrenia the rate of progression of hippocampal abnormality may be slower than at later stages of illness, or that volume changes have already occurred (Lawrie et al., 1999).

Our analysis of longitudinal shape change suggests that volume loss does not occur universally across the hippocampus. The surface map findings showed patchy areas of surface contraction or expansion. These findings are plausible as prior post-mortem studies of schizophrenia have found the pathology to be focal and much less evenly distributed throughout the medial temporal lobe structures (Harrison, 2004; Heckers and Konradi, 2010). In addition, given the anatomy of the hippocampus and its composition by distinct cytoarchitectural regions it stands to reason that different areas would be affected, by different degrees and sequence (Mai et al., 1997). Visual observation of surface maps suggested that HAL have larger regions of longitudinal surface contractions than either OLZ or controls. A larger region of surface contraction with HAL was supported by a left shift of the cumulative distribution of the hippocampal vertex slopes in the HAL group compared to other groups. Differences in the percentage of negative slopes between HAL and OLZ, however did not reach significance. However, when the percentages of *large magnitude* slopes were compared, significant group differences were observed, with HAL having more large magnitude negative slopes. This indicates that with both OLZ and HAL treatment various regions of the hippocampus contract over time, however the magnitudes of these surface contractions differ.

Olanzapine-associated effects could reflect a therapeutic effect of treatment (in preventing a disease-associated volume loss) and/or a neuroplastic adaptation to chronic treatment, such as has been seen in the caudate and lenticular nuclei (Chakos et al., 1994, 1998; Roberts et al., 1995) and in prefrontal cortex (Selemon and Goldman-Rakic, 1999). Antipsychotic drugs have been found to have a variety of effects on brain structure and volume both protective or enhancing (Konradi and Heckers, 2001) and associated with volume reduction (Dorph-Petersen et al., 2005; Ho et al., 2011). Specific atypical antipsychotic drugs (particular clozapine and OLZ) have been reported to have various actions that could enhance cellular resilience and ameliorate the pathophysiology of schizophrenia (Lieberman et al., 2008). These include the antagonism of the effects of *N*-methyl-d-aspartate receptor antagonists (Olney and Farber, 1995; Duncan et al., 2000), increased expression of tropic factors (Bai et al., 2003; Fumagalli et al., 2003; Marx et al., 2003), and stimulation of neurogenesis (Wakade et al., 2002; Halim et al., 2004). The effects of antipsychotics may also result from alterations in blood flow and metabolism within the hippocampus as opposed to neuronal changes (Miller et al., 1997b; Cohen et al., 1999; Molina et al., 2003; Lahti et al., 2004). As the hippocampal surface vertex slopes of untreated psychotic patients are unknown, it is unclear if HAL prevents surface contraction to any degree. However, hippocampal volume loss has been associated with quetiapine in first-episode psychosis over 6 months, especially at higher doses (Ebdrup et al., 2010).

There are potential limitations to our study findings. Despite the highly significant group difference in the number of large negative hippocampal surface vertex slopes, the maps of individual surface slopes did not reveal any vertices with differences between OLZ and HAL treatment after 5% FDR correction. However, these seemingly contradictory findings imply that hippocampal regions affected are likely non-specific, and potentially vary between individuals. If antipsychotic treatment affects different surface vertices in different individuals, it would be difficult to detect a region of consistent slope differences in OLZ vs. HAL. This is supported by visual observation of individual subjects’ hippocampal surfaces, which showed considerable within-group variability in the surface locations of negative slopes. A difference in the number of large magnitude negative slopes between OLZ and HAL groups indicates that there is however an overall differential treatment effect. Future studies would be required to assess whether underlying patient specific factors that may influence hippocampal response (e.g., substance use, underlying medical conditions, genetic risk factors, or specific clinical phenotypes) result in differential treatment effects of antipsychotics on hippocampal structure. Such patient data were not available for this study. Our study also had slightly more females in the control group, compared to the psychotic group, although controlling for gender did not significantly affect baseline hippocampal differences in our study (Gur et al., 1991; Murphy et al., 1996). This would not however influence the primary results of our studies comparing OLZ and HAL as these groups did not differ significantly by gender. Our results may also have been affected by measurement variability from the multiple scanners used. However rigorous quality control efforts where applied to establish reliability of scanner measurements, which would minimize such effects (Styner et al., 2002). Furthermore, allocation to treatment group occurred randomly across sites; thus any potential site effects would be expected to equally affect both treatment groups. Hippocampal shape change may also have been influenced by longitudinal changes in clinical symptoms, such as psychosis, depression, anxiety, or cognitive abnormalities. Structural findings may thus have been in part driven by differences in response rates between the two treatment groups. However these clinical measures were not available for the current study, thus the relationships were not investigated.

In summary, compared to the hippocampus of patients treated with OLZ, those of patients treated with HAL had a larger proportion of large magnitude negative slopes, indicating greater contraction of a certain portion of their surfaces over time. Further studies will be required to evaluate the significance of these hippocampal regions in the pathogenesis and pathophysiology of schizophrenia and the relationship between the magnitude of surface contraction and clinical or cognitive changes in patients with psychotic disorders.

## Conflict of Interest Statement

The authors declare that the research was conducted in the absence of any commercial or financial relationships that could be construed as a potential conflict of interest.
